# Concurrent *Cryptosporidium parvum* outbreaks: molecular characterisation supporting epidemiological investigations leads to identification of different implicated food items, Sweden, 2019

**DOI:** 10.2807/1560-7917.ES.2025.30.26.2400643

**Published:** 2025-07-03

**Authors:** Ioana Bujila, Marie Jansson-Mörk, Joanna Nederby-Öhd, Anette Hansen, Mats Lindblad, Karolina Fischerstöm, Matilda Bragd, Ingela Hall, Nilla Lindroos, Caroline Rönnberg, Moa Rehn, Jessica Beser

**Affiliations:** 1 Department of Microbiology, Public Health Agency of Sweden, Solna, Sweden; 2 Department of Communicable Disease Control and Health Protection, Public Health Agency of Sweden, Solna, Sweden; 3 ECDC Fellowship Programme, Field Epidemiology path (EPIET), European Centre for Disease Prevention and Control (ECDC), Stockholm, Sweden; 4 Department of Communicable Disease Control and Prevention, Region Stockholm, Stockholm, Sweden; 5 Swedish Food Agency, Uppsala, Sweden; 6 Department of Communicable Disease Control and Prevention, Region Jönköping, Jönköping, Sweden; 7 Department of Communicable Disease Control and Prevention, Region Halland, Halmstad, Sweden; 8 Department of Medicine, Solna, Karolinska Institutet, Stockholm, Sweden; 9 ECDC Fellowship Programme, Public Health Microbiology path (EUPHEM), European Centre for Disease Prevention and Control (ECDC), Stockholm, Sweden

**Keywords:** Protozoan Infections, Cryptosporidiosis, Epidemiology, Molecular Typing, Disease Outbreak, Cohort Study, Case-case Study, Source Tracing, Foodborne Outbreak, Diarrhoea

## Abstract

In Sweden, an increase in the number of notified cases of cryptosporidiosis was observed 1 October–31 December 2019 (462 domestic cases). Although a single national outbreak was initially suspected, molecular and epidemiological analyses revealed two concurrent national outbreaks and three local outbreaks. *Cryptosporidium parvum* subtype IIdA22G1c and IIdA24G1 were identified as the cause of the national outbreaks and subtype IIdA20G1e and IIdA21G1 as the cause of the local outbreaks. A case-case study comparing exposures in IIdA22G1c to IIdA24G1 revealed that cases with subtype IIdA22G1c (n = 48) were associated with consumption of a fresh fruit-and-vegetable juice (adjusted odds ratio (aOR) = 17; 95% confidence interval (CI): 1.8–169; p = 0.002). In the local outbreaks with subtype IIdA20G1e and IIdA21G1, cohort studies suggested that cases were associated with consumption of salads. Several coinciding outbreaks with different *C. parvum* subtypes explained the increase of cryptosporidiosis, and molecular typing was crucial in guiding relevant cross-disciplinary collaboration.

**Key public health message**

**What did you want to address in this study and why?**

Cryptosporidiosis is a diarrhoeal disease caused by the protozoan parasite *Cryptosporidium*. We aimed to investigate the cause of an increase in cryptosporidiosis cases in Sweden in 2019. Further, we aimed to find the source of any potential outbreak and implement measures to contain the outbreak.

**What have we learnt from this study?**

By combining microbiological and epidemiological methods, we could conclude that the increase was caused by several foodborne outbreaks occurring concurrently. We suspected consumption of leafy green vegetables as the source of the outbreaks. The trace-back investigation of fresh produce was challenging.

**What are the implications of your findings for public health?**

Leafy green vegetables warrant further awareness. Specifically, the findings suggest that consumption of unpasteurised juice containing contaminated leafy green vegetables can cause cryptosporidiosis which needs to be communicated to producers of such beverages, along with recommendations for preventive measures.

## Background

*Cryptosporidium* is a protozoan parasite that can cause disease in humans and animals. In humans, the infection usually presents with self-limiting diarrhoea, but the disease can be severe, especially in individuals with a weakened immune system or who are malnourished [1]. The largest known outbreaks of cryptosporidiosis in Europe occurred in Sweden in 2010 and 2011 due to contamination of the municipal drinking water with *Cryptosporidium hominis* [2,3]. Foodborne outbreaks of cryptosporidiosis are frequently reported in Europe [4,5]. In Sweden, more than 30 foodborne outbreaks caused by *Cryptosporidium* spp. have been identified between 2007 and 2024 [6-8]. No outbreaks due to contaminated drinking or recreational water have been reported in Sweden since 2016 [6-8]. Annual seasonal peaks of cryptosporidiosis occur during late summer months and autumn in European countries [7,9]. Hence, outbreaks during this period can initially be difficult to detect.

## Outbreak detection

Beginning in August 2019, an increase in the notification rate of cryptosporidiosis was observed in several regions of Sweden, with an escalation in the beginning of October 2019. In addition, an increase in inquiries about gastrointestinal symptoms to the national healthcare call line 1177 indicated that an outbreak might be ongoing. Inquiries through 1177 have previously been a signal of ongoing outbreaks in Sweden [2].

An outbreak investigation team was established and included representatives from the Public Health Agency of Sweden (PHAS), the Swedish Food Agency (SFA) and the affected regional departments of communicable disease control (CDC).

Here, we describe the investigation of the increase and the value of molecular characterisation, which distinguished coinciding outbreaks of different subtypes of *C. parvum*. Further, molecular characterisation together with epidemiological investigations identified possible sources of contamination.

## Methods

### Surveillance of cryptosporidiosis

Cryptosporidiosis has been a notifiable disease in Sweden since 2004, and cases are reported to the Swedish notification system for notifiable communicable diseases (Sminet). A suspected case is defined as a person presenting clinical symptoms consistent with a *Cryptosporidium* spp. infection, combined with an established epidemiological link. A confirmed case is a laboratory-diagnosed case of *Cryptosporidium* spp. The regional CDC departments also perform event-based surveillance and are responsible for local and regional outbreak investigations and are encouraged to distribute web-based trawling questionnaires to cases.

In addition, between 2018 and 2022, a national microbiological surveillance programme for cryptosporidiosis was running, where *Cryptosporidium*-positive samples from domestically infected cases were routinely sent to PHAS for molecular characterisation.

### Primary diagnostics and molecular characterisation

Primary diagnostics were performed at regional clinical microbiological laboratories using real-time PCR and/or to a lesser extent by microscopy using modified Ziehl-Neelsen staining. Positive samples (extracted DNA or faeces) of domestic cases were sent to PHAS for molecular characterisation.

Due to the large number of positive samples and limited resources, only a subset was selected for molecular characterisation. Samples were primarily selected from different regions to assess the geographical distribution, with additional samples included from regions with a stronger suspicion of an outbreak. Priority was also given to samples from cases that had completed a trawling questionnaire.

Species identification and subtyping were performed as described in Bujila et al. [6], based on amplification and sequencing of the small subunit rRNA (*ssu rRNA*) gene as described by Xiao et al. [10,11] and the 60 kDa glycoprotein (*gp60*) gene described by Alves et al. [12].

### Outbreak case definitions

The case definitions used in the national outbreak and the local outbreaks are described in Box.

Box **Case definitions in the outbreaks of cryptosporidiosis, Sweden, 2019**

**National outbreaks:**

*Confirmed case*

• A person with laboratory-confirmed cryptosporidiosis

AND

• Onset of disease or date of sampling (if onset date was missing) 1 October–31 December 2019

AND

• No recent travel.

**Local outbreaks:**

*Suspected case*

• Region Västerbotten: A person with gastrointestinal symptoms 13–24 December 2019 and attending a lunch in the workplace canteen 11–12 December 2019,

• Region Kronoberg: A person with diarrhoea and/or at least two of the following symptoms: vomiting, abdominal pain, fever, nausea or headache 20–30 December 2019 and attending the Christmas dinner on 18 December 2019,

• Region Stockholm: A person with gastrointestinal symptoms 15–25 December 2019 and attending the Christmas dinner on 13 December 2019

*Confirmed case*

• Suspected case with laboratory-confirmed cryptosporidiosis.

### Epidemiological investigations

Information on all laboratory-confirmed cryptosporidiosis cases was collected from Sminet, including age, sex (male/female), region, date of disease onset and sampling date.

In the national outbreak investigation, confirmed cases were asked by the regional CDC departments to complete a web-based trawling questionnaire focused on food and environmental exposures. Results from molecular characterisation were used to design a case-case study, comparing cases from two larger clusters of two different *C. parvum* subtypes in terms of exposure based on data from the trawling questionnaire.

Through event-based surveillance, three different regions were informed about possible local outbreaks occurring after a Christmas lunch served in a workplace and two different Christmas dinners. The CDC departments of Region Västerbotten, Region Stockholm and Region Kronoberg initiated separate outbreak investigations and sent out outbreak-specific questionnaires including questions about what was served during the lunch or dinners. In Region Västerbotten, the questionnaire was sent out to all employees of the workplace where the Christmas lunch had been served in the canteen. In Stockholm and Kronoberg, the questionnaires were sent out to all dinner participants. Cohort studies were conducted using the outbreak-specific questionnaires.

### Statistical analyses

In the case-case study, for each food item, the options ‘yes’ and ‘probably’ were categorised as exposed, and options ‘no‘ or ‘probably not‘ were categorised as unexposed. The option ‘do not know’ was treated as missing. Some food items, such as different types of salads, were grouped as one exposure in the analysis. From open-ended questions about the place of purchase of the food items, specific retailers were identified. In the logistic regression analysis, cluster (1/0; IIdA22G1c/IIdA24G1) was the dependent variable. Initially, for food items and retailers to which at least 50% of the cases in either cluster were exposed, adjusted odds ratios (aOR) were estimated with 95% confidence intervals (CI) and p values. Next, all food items and retailers with a p value < 0.2 were included in a multivariable analysis. If a food item was eligible for the final model by itself or as a grouped exposure, the exposure with the highest log-likelihood was selected. The final model used manual forward inclusion, where exposures were kept in the model if they had a p value < 0.05 or changed the estimate of another exposure with more than 15%, i.e. being considered a confounder. All models were adjusted for age, sex (as a binary variable) and region (Stockholm/other regions). Stata version 16.0 was used in the analyses (https://www.stata.com).

In the cohort studies of the three local outbreaks, questionnaires were used to identify cases and estimate attack rates (AR). Items with a risk ratio (RR) > 1, p value ≤ 0.2 and with at least 10 exposed cohort members were included in a stratified analysis to identify effect modification (i.e. where the significance of the exposure depended on the value of the main suspected food item). Food items not affected by effect modification were included in a multivariable analysis using Poisson regression estimating adjusted risk ratios (aRR) with 95% CI and p values. Stata version 16 was used in the analyses.

## Results

### Descriptive epidemiology

**Figure 1:**
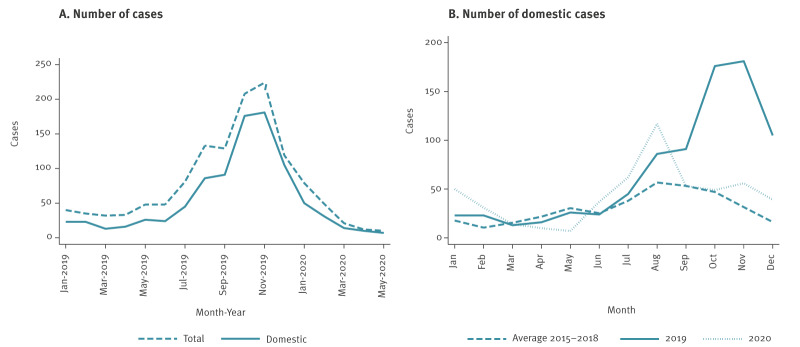
Number of notified laboratory-confirmed cases of cryptosporidiosis in total and number of domestic cases, by month, Sweden, 2019–2020

**Figure 2:**
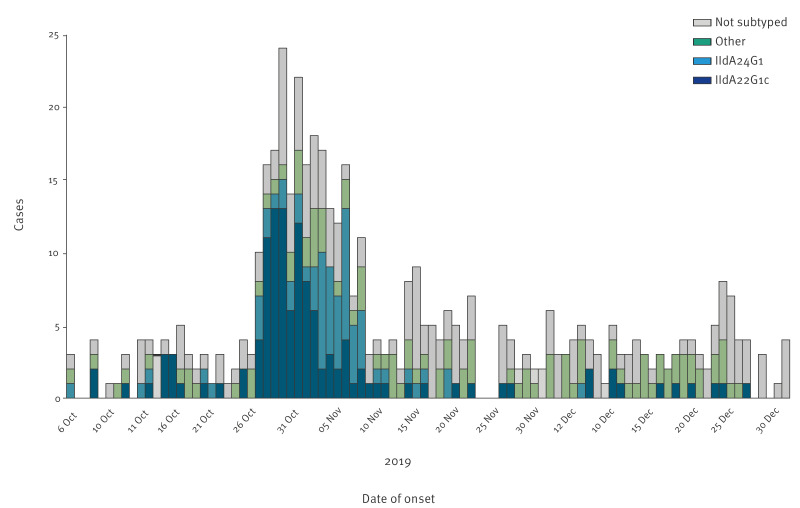
Timeline of confirmed cases of cryptosporidiosis, by subtype^a^, Sweden, 1 October–31 December 2019, (n = 462)

There was a clear surge above the expected in the notification rate of cryptosporidiosis October–December 2019 (Figure 1). In total, 462 domestic cases with onset of disease or date of sampling 1 October–31 December (Figure 2) were reported to Sminet from 20 of 21 regions in Sweden. Region Stockholm notified the highest number of cases (n = 173; 37%) followed by Västra Götaland (n = 61; 13%) and Östergötland (n = 59; 13%). Among cases, 253 (55%) were female and 209 (45%) were male. The age range was 0–87 years with a median age of 38 years (interquartile range (IQR): 28–49 years).

If information on the date of onset was missing, sampling date was used.

^a^ The two most common subtypes included.

### Molecular characterisation

Molecular characterisation was performed for 296 (64%) samples. Species determination was successful for 289 (98%) samples of which 283 (98%) were *C. parvum*. In three samples, *C. mortiferum* was identified, and *C. hominis*, *C. cuniculus* and *C. felis* was identified in one sample each. Subtyping with *gp60* was successful for 278 (98%) of the *C. parvum* samples (Table 1). In total, 24 different *C. parvum* subtypes were identified during the outbreak period of which IIdA22G1c (n = 123; 44%), IIdA24G1 (n = 65; 23%), IIdA20G1e (n = 24; 9%) and IIdA21G1 (n = 20; 7%) were the most common. Cases with the two most frequent *C. parvum* subtypes are also presented in Figure 2.

**Table 1:** *Cryptosporidium parvum* subtypes and GenBank accession numbers of confirmed cases of cryptosporidiosis, Sweden, 1 October–31 December 2019 (n = 278)

Subtype	n	GenBank accession number
IIdA22G1c	123	FJ917374
IIdA24G1	65	JQ028865
IIdA20G1e	24	JQ028866
IIdA21G1	20	OL598545
IIdA19G1	6	DQ280496
IIaA22G1R1	5	JX183806
IIaA14R1	5	JX183797
IIdA23G1	4	FJ917376
IIdA25G1	4	JX043492
IIaA16G1R1b	3	EU647727
IIaA18G1R1b_variant	3	KT895369
IIaA15G2R1	3	AF164490
IIaA13R1	2	KU852702
IIaA14G1R1r1	1	KU852703
IIaA17G1R1c	1	JX183801
IIaA17G1R1c_variant	1	AF403168
IIaA17R1	1	JX183800
IIaA20G1R1	1	JX183804
IIaA21G1R1	1	FJ917373
IIaA23G1R1	1	KC995126
IIdA16G1	1	JX183808
IIdA16G1b	1	FJ917372
IIdA18G1	1	KU852709
IIdA26G1b	1	JX183811

These cases have also been included in the overall summary of the molecular surveillance 2018–2022 in Sweden [6].

### Case-case study

*Cryptosporidium parvum* subtype IIdA22G1c was detected in samples from cases residing in 10 regions and subtype IIdA24G1 in 14 regions. In total, 65 cases (53%) with subtype IIdA22G1c and 45 cases (69%) with subtype IIdA24G1 completed the trawling questionnaire and were thus included in the case-case study. The distribution of cases in the study per subtype, region, age group and sex are shown in Table 2.

**Table 2:** Characteristics of cases with *Cryptosporidium parvum* subtype IIdA22G1c (n = 65) and IIdA24G1 (n = 45) included in the case–case study, by region, sex and age, Sweden, 1 October–31 December 2019

Characteristics	Subtype IIdA22G1c (n = 65)		Subtype IIdA24G1 (n = 45)	
	n	%	n	%
Region				
Stockholm	21	32	3	7
Other	44	68	42	93
Sex				
Female	41	63	30	67
Male	24	37	15	33
Age (years)	Median	IQR	Median	IQR
	39	30–46	35	30–50

IQR: Interquartile range.

Several cases infected with *C. parvum* subtype IIdA22G1c and interviewed by one regional CDC reported that they had been drinking fresh fruit-and-vegetable juice. Thus, we considered that fresh fruit-and-vegetable juice could have been the vehicle in the cluster caused by *C. parvum* subtype IIdA22G1c, and another vehicle could have been in the cluster caused by *C. parvum* subtype IIdA24G1. The exposures to which at least 50% of cases with either subtype IIdA22G1c or IIdA24G1 were exposed are shown in Supplementary Table 1.

Not all 110 cases who responded to the questionnaire replied to all questions and, therefore, the final model included 84 cases i.e. IIdA22G1c (n = 48) and IIdA24G1 (n = 36). The final model showed that cases with subtype IIdA22G1c were more likely to have consumed fresh unpasteurised fruit-and-vegetable juice from Brand A (aOR = 17; 95% CI: 1.8–169) and more likely to have eaten at cafés (aOR = 5.7; 95% CI: 1.4–23) compared with the cases with subtype IIdA24G1 (Table 3).

**Table 3:** Adjusted odds ratios of cryptosporidiosis cases with subtype IIdA22G1c (n = 48) compared with cryptosporidiosis cases with subtype IIdA24G1 (n = 36) in a final multivariable model of case-case study, Sweden, 1 October–31 December 2019

Exposure	Exposed IIdA22G1c (%)	Exposed IIdA24G1 (%)	aOR	95% CI	p value
Fresh fruit-and-vegetable juice, Brand A	52	2.8	17	1.8–169	0.002
Eating at cafés	65	31	5.7	1.4–23	0.010
Spinach	67	42	3.9	0.9–16	0.050
Iceberg lettuce	48	69	0.12	0.1–0.6	0.003
Salad buffet from grocery store	33	50	0.08	0.01–0.4	0.001

aOR: adjusted odds ratio; CI: confidence interval.

Conversely, cases with subtype IIdA22G1c were less likely to have eaten salad buffet from a grocery store (aOR = 0.08; 95% CI: 0.01–0.4) or iceberg lettuce (aOR = 0.12; 95% CI: 0.1–0.6) compared with cases with subtype IIdA24G1.

### Cohort studies

In Region Västerbotten, questionnaires from 107 respondents were included in the analysis. Of these, three (3%) were laboratory-confirmed and 46 (43%) were suspected cases according to the outbreak case definition. Individuals who had eaten a salad containing kale, apples, onion and sour cream in the workplace canteen were more likely of becoming ill compared with those not eaten the salad (aRR = 3.7; 95% CI: 1.3–11). More details are presented in Supplementary Table 2.

In Region Kronoberg, questionnaires from 63 respondents were included in the analysis. Of these, four (6%) were laboratory-confirmed and 21 (33%) were suspected cases according to the outbreak case definition. Individuals who had eaten kale salad at the Christmas dinner were more likely (aRR = 15; 95% CI: 1.9–117) of becoming ill than those not eaten kale salad. More results are presented in Supplementary Table 3.

In Region Stockholm, questionnaires from 37 respondents were included in the analysis. Of these, two (5%) were laboratory-confirmed and 19 (51%) were suspected cases according to the outbreak case definition. Individuals who had eaten salad containing kale and apples (aRR = 4.0; 95% CI: 0.9–18), cured salmon (aRR = 2.6; 95% CI: 1.4–4.8) or hard rye bread (aRR = 2.3; CI: 1.4–3.8) were more likely of becoming ill compared with unexposed, presented in Supplementary Table 4.

Samples from two confirmed cases from each local outbreak were characterised. The samples from Region Västerbotten were *C. parvum* subtype IIdA20G1e and the samples from Region Kronoberg and Stockholm were *C. parvum* subtype IIdA21G1.

### Trace-back investigations

Spinach was the only ingredient exclusively added to the implicated contaminated fresh fruit-and-vegetable juice from Brand A, compared with other juices from the same brand. Hence, it was suspected to be the contaminated food item in the juice. An attempt to trace back spinach was made but we could not identify one single batch of spinach. During the period when possibly contaminated batches were produced, the production company in Sweden received spinach from a company in the Netherlands that sold spinach grown both in the Netherlands and Spain, and the country of origin could not be identified.

As kale was the major component of the suspected contaminated salads in two local outbreaks and identified as a possible source also in the third outbreak, an attempt to trace back kale was made. Kale served at the three occasions originated from different growers in southern Sweden. There was no common producer for the kale served at the two different Christmas dinners in Region Kronoberg and Stockholm, even though the samples from the cases had the same subtype of *C. parvum*. However, all growers producing the suspected kale were located within a maximum distance of 40 km and contacted by their competent authorities. All growers were specialised in vegetable production, and there were no livestock at or in the close vicinity of the farms. All farms used mineral fertilisers, and water for irrigation came from deep-drilled wells. We could not identify a likely source of contamination on any of the kale farms. No food items from any of the occasions were left for microbiological analysis at the time of the investigation.

### Outbreak response measures

The Public Health Agency of Sweden continuously posted updates about the outbreaks on its website between 13 November 2019 and 24 February 2020. On 24 February 2020, the outbreak was declared over, and the investigation was closed. An item was also posted in the Epidemic Intelligence Information System (EPIS) on 11 December 2019 to alert other European countries about the increase of the two *C. parvum* subtypes IIdA22G1c and IIdA24G1. No other country reported cases related to these outbreaks. None of the implicated food items from any of the outbreaks was available at retail at the time of the outbreak investigation due to short shelf life. Therefore, no recalls or withdrawals could be done. The manufacturer of the fresh fruit-and-vegetable juice as well as the kale growers were informed about the incidents with recommendations of preventive measures. These included additional quality control procedures for the manufacturer of the juice and general hygiene practices, along with information about potential sources of contamination for the kale growers. Also, PHAS informed on their website on the implications of consuming fresh unpasteurised fruit-and-vegetable juice. These beverages are not heat-treated and may therefore contain pathogenic microorganisms.

## Discussion

Molecular characterisation in the outbreak investigation revealed that several outbreaks coincided during the autumn–winter of 2019 in Sweden. Based on the epidemiological findings, various leafy green vegetables were the suspected source of contamination. Contaminated leafy green vegetables are recurrently causing outbreaks of cryptosporidiosis. The largest reported foodborne outbreaks of cryptosporidiosis in Europe occurred in Finland in 2012 with > 250 cases associated with consumption of frisée salad, and in the United Kingdom (UK) in 2012 with > 300 cases associated with consumption of pre-cut salad leaves and in 2015 with > 400 cases associated with salad from a coffee shop chain [5,13,14]. Most outbreaks of cryptosporidiosis investigated in Sweden have been associated with contaminated food items [6,8,15] and the majority attributed to infection with *C. parvum*. In 2010, an outbreak with 96 suspected cases occurred after a conference dinner, and a cohort study identified herbs on a meat dish as the probable source of infection [7,16]. In 2014, an outbreak with 83 suspected cases and 23 laboratory-confirmed cases of *C. parvum* subtype IIdA22G1 was linked to eating at a specific restaurant where parsley was the suspected source of infection [7]. In 2015, another outbreak occurred at a conference with 5,000 participants. The investigation identified 83 cases of which nine were laboratory-confirmed with *C. parvum* subtype IIaA17G1R1 [7]. In addition, foodborne outbreaks with ≤ 30 cases have been described [6-8,15,16].

The identification of fresh fruit-and-vegetable juice as the likely source of infection for the national outbreak of *C. parvum* subtype IIdA22G1c was the first time a beverage was reported as a possible vehicle of *Cryptosporidium* in Sweden. In other European countries, outbreaks have been associated with beverages. Milk was identified as the source in an outbreak in England in 2012, and unpasteurised apple juice in Norway in 2018 [17,18]. Taken together, this highlights the risk of cryptosporidiosis associated with unpasteurised beverages. Spinach in the fresh fruit-and-vegetable juice was the suspected contaminated food item. Eating at a café was also associated with infection, with a lower aOR. The contaminated juice could have been served at cafés, as this is a popular brand of juice served at cafés, however, we could not further investigate this. Subtype IIdA22G1c is one of the most common *C. parvum* subtypes in Sweden [6]. In 2016, 40 suspected cases with this subtype fell ill after having visited a zoo and waterpark. In 2022, this subtype was also identified in an outbreak with 75 suspected cases at an upper secondary school where contaminated salad buffet was suspected as the source of infection [6].

The source of the second national outbreak, which was caused by *C. parvum* subtype IIdA24G1, could not be further investigated due to the start of the COVID-19 pandemic, although the investigation indicated salad buffet from grocery stores and iceberg lettuce. Subtype IIdA24G1 is also among the most common *C. parvum* subtypes in Sweden [6]. It has previously been associated with foodborne and zoonotic outbreaks, in Sweden and in other European countries, one of them being the previously mentioned large outbreak in the UK in 2015 [16,19,20].

During the outbreak period, a few cases involving other species, including *C. mortiferum*, *C. hominis*, *C. cuniculus* and *C. felis*, were identified alongside *C. parvum*. This aligns with previous findings of the epidemiology of cryptosporidiosis in Sweden [6,8,21].

In the local outbreaks, only a few cases were laboratory-confirmed, so the involvement of another pathogen in addition to *C. parvum* causing the symptoms cannot be ruled out. In all three investigations, salads including kale were identified as a possible vehicle of infection. In Region Stockholm, salmon and hard rye bread were also identified as possible sources. Although not statistically significant, kale was considered a more likely source than salmon and hard rye bread. As kale was the only food item implicated in all three local outbreaks occurring within 8 days, the trace-back investigation focused on kale. After the outbreaks described here, another outbreak was investigated in Sweden in 2023 where kale was suspected to be the contaminated food item [22]. Trace-back investigations identified kale growers from Sweden, Belgium and Spain but no particular grower could be identified at that later time point either, emphasising the difficulties to trace back fresh produce. Foodborne outbreaks of cryptosporidiosis are in general challenging to investigate in addition to difficulties in trace-back investigations. The pathogen has a relatively long incubation period and by the time an outbreak is identified, there are often no food (or beverage) items left to collect for microbiological analyses. Further, if collection of food items is successful, analysis of *Cryptosporidium* spp. is quite challenging on these matrices [23].

Contamination with *Cryptosporidium* oocysts can occur at any stage of food processing. Potential sources include exposure to manure, contaminated soil or water, as well as direct contamination from animals or infected food handlers [24]. Given Sweden's recurrent foodborne outbreaks, two new initiatives have been launched to investigate the impact of animals and extreme weather on *Cryptosporidium* spp. contamination of crops [25,26], which could provide more insight into the primary causes of contamination, enabling the implementation of appropriate preventive measures.

As outbreaks of cryptosporidiosis in Sweden often have similar transmission modes, molecular characterisation is essential. A limitation of the typing tool used in this study is that *gp60* may lack sufficient discrimination for certain common subtypes. To address this, a more in-depth molecular approach, as described by Robinson et al. in 2022, could serve as a valuable complement for case assignment and potentially source attribution, particularly if food samples are available for analysis [27,28].

The seasonal peak commonly observed in Sweden in late summer may result from several contributing factors, such as increased outdoor activities, time spent in forests foraging for berries and mushrooms, more frequent visits to petting zoos and farms and higher consumption of vegetables and salads. In 2019, the surge began in August, i.e. earlier than the period investigated. Thus, other outbreaks might have been undetected in August and September or that one of the national outbreaks started earlier.

Epidemiological limitations in this investigation include risk of recall bias possibly leading to misclassification of exposures. The approach of a case-case study rather than a case-control study was chosen due to the relatively long time between the infection and the collection of data and the multiple simultaneously ongoing outbreaks. Since answers from trawling questionnaires were already being collected and the cases of one outbreak could be distinguished from cases of the other outbreak by molecular characterisation, we could use already collected data and conduct a case–case study. Case-case study based on one trawling questionnaire was used successfully in an investigation of an outbreak of *Salmonella* in Sweden [29].

The recent investigations of increases of cryptosporidiosis highlight the importance of strengthening the surveillance of cryptosporidiosis in Europe [30-32]. This could be achieved by making the disease notifiable in all European countries, reinforcing event-based surveillance and implementing molecular surveillance programs. Additionally, surveillance is influenced by the adoption of more sensitive diagnostic methods and the increasing use of multiplex assays capable of detecting multiple agents simultaneously. These advancements reduce the reliance on clinicians' specific choices for analysis, important as *Cryptosporidium* spp. often go under-recognised [33,34].

## Conclusion

The data presented here stress the importance of strengthening the surveillance of cryptosporidiosis in Europe. A combined approach of molecular and epidemiological investigations in addition to a One Health approach is crucial to investigate and manage outbreaks. To ensure food safety we emphasise the importance of increasing awareness about leafy green vegetables as vehicles for *C. parvum* outbreaks, also when included in unpasteurised beverages.

## Data Availability

Data are available upon reasonable request. Some data cannot be accessed for reasons of confidentiality and personal data protection.

## Supplementary Data

301000 Supplementary Material
