# Supplementary material for: Concurrent Cryptosporidium parvum outbreaks: molecular characterisation supporting epidemiological investigations leads to identification of different implicated food items, Sweden, 2019
Source: Euro Surveill. 2025 Jul 3;30(26):2400643. doi: 10.2807/1560-7917.ES.2025.30.26.2400643 (PMC12231374; doi:10.2807/1560-7917.ES.2025.30.26.2400643)
Supplement: Supplementary Material [file 24-00643_BESER_Supplement.pdf]

This supplementary material is hosted by Eurosurveillance as supporting information alongside the article ' Concurrent Cryptosporidium parvum outbreaks: molecular characterisation supporting epidemiological investigations leads to identification of different implicated food items, Sweden, 2019', on behalf of the authors, who remain responsible for the accuracy and appropriateness of the content. The same standards for ethics, copyright, attributions and permissions as for the article apply. Supplements are not edited by Eurosurveillance and the journal is not responsible for the maintenance of any links or email addresses provided therein.

SUPPLEMENTARY TABLES

Supplementary Table 1. Number and percentage exposed, adjusted odds ratios (aOR) and 95% CI of cryptosporidiosis cases with subtype IIdA22G1c compared to cases with subtype IIdA24G1 during the outbreak period 1 October – 31 December 2019 in Sweden.

Exposure	Cases with subtype IIdA22G1c (n=65)		Cases with subtype IIdA24G1 (n=45)		Model for each exposure[1]		
	n[2]	%[3]	n[2]	%[3]	aOR[4]	95% CI	p value
Fresh fruit- and vegetable juice, brand A	32	50.8	1	2.3	38.5	4.8-310	<0.001
Fresh juice	44	69.8	8	18.6	8.7	3.3-22.8	<0.001
Juice, all sorts	45	71.4	11	25.6	6.3	2.6-15.7	<0.001
Spinach	42	70	18	43.9	2.4	1.0-5.7	0.05
Apple	49	79.0	29	65.9	2.3	0.9-6.0	0.08
Café	38	61.3	15	35.7	2.0	0.8-4.8	0.12
Mixed lettuce	46	75.4	22	53.7	2.0	0.8-4.8	0.14
Restaurants (other than hamburger restaurants)	43	72.9	22	53.7	1.6	0.6-3.9	0.33
Arugula	33	55.9	17	41.5	1.5	0.7-3.6	0.32

Cucumber	55	85.9	37	84.1	1.3	0.4-4.2	0.63
Tomato	55	85.9	36	81.8	1.2	0.4-3.6	0.8
Other fruits	33	54.1	20	51.3	1.1	0.4-2.5	0.92
Restaurant - pizzeria	30	52.6	15	37.5	1.1	0.4-2.7	0.85
Ready-to-eat salad	47	73.4	30	69.8	0.9	0.4-2.3	0.84
Retailer brand A	52	85.3	36	87.8	0.8	0.2-2.8	0.73
Carrot	43	66.2	29	65.9	0.8	0.4-1.9	0.65
Pepper	40	65.6	30	68.2	0.8	0.3-1.8	0.54
Red onion	33	53.2	30	68.2	0.7	0.3-1.5	0.32
Yellow onion	45	72.6	34	77.3	0.6	0.3-1.6	0.34
Lettuce (all sorts)	43	68.3	31	75.6	0.5	0.2-1.3	0.14
Minced meat (beef or mix of beef and pork)	45	69.2	37	84.1	0.4	0.2-1.1	0.07
Iceberg lettuce	30	49.2	28	68.3	0.4	0.2-1.0	0.04
Salad buffet from grocery store	17	30.9	21	52.5	0.1	0.03-0.3	<0.001

[1]Each exposure model adjusted for sex, age group and county
[2] Number exposed
[3] Percentage exposed of those who answered
[4] Drinking water (own well or municipal drinking water) was excluded from further analysis, 98% of cases in subtype IIdA22G1c and 100% of cases in subtype IIdA24G1 were exposed to municipal drinking water.

Supplementary Table 2. Total number, number of cases and attack rate (AR) for individuals exposed and unexposed to different dishes served in the canteen 11-12 December in Västerbotten County, Sweden 2019, risk ratio (RR) and adjusted RR (aRR) with 95% confidence interval (CI) and p-value (Walds test) from univariable and multivariable analyses.

**Exposure**	**Exposed**			**Unexposed**			**Univariable model**			**Multivariable model**		
	Total	Cases	AR %	Total	Cases	AR %	RR	95 % CI	p value	aRR	95 % CI	p value
Kale salad	36	33	91.7	19	4	21.1	4.4	1.8–10.4	<0.001	3.66	1.23–10.7	0.018
Liver paté	15	14	93.3	44	26	59.1	1.6	1.2–2.1	0.014	1.27	0.6–2.6	0.52
Vegetarian Christmas ham	17	15	88.2	46	28	60.9	1.5	1.1–1.9	0.038	1.27	0.6–2.6	0.52
Fennel salad	24	20	83.3	28	13	46.4	1.8	1.2–2.8	0.006	NA[a]		
Raw spiced salmon	59	42	71.2	5	2	40.0	1.8	0.6–5.3	0.149	NA		
Potato	55	40	72.7	9	4	44.4	1.6	0.8–3.5	0.09	NA		
Pickled Herring, sauce A	46	35	76.1	16	8	50.0	1.5	0.9–2.6	0.051	NA		
Mimosa	18	15	83.3	38	22	57.9	1.4	1.0–2.0	0.06	NA		
Sauce	42	32	76.2	22	12	54.6	1.4	0.9–2.1	0.076	NA		
Pickled herring, sauce B	46	34	73.9	15	8	53.3	1.4	0.8–2.3	0.135	NA		
Beetroot	38	28	73.7	24	14	58.3	1.3	0.9–1.9	0.208	NA		
Ham	50	36	72.0	12	7	58.3	1.2	0.7–2.1	0.356	NA		
Egg	54	38	70.4	7	4	57.1	1.2	0.6–2.4	0.477	NA		

[a] Effect modification was identified in the stratified analysis of fennel salad (strata by exposure of winter salad) and fennel salad was therefore excluded from the multivariable analysis.

Supplementary Table 3. Total number, number of cases and attack rate (AR) for individuals exposed and unexposed to different dishes served at the dinner 18 of December in Kronoberg County, Sweden 2019, risk ratio (RR) and adjusted RR (aRR) with 95% confidence interval (CI) and p-value (Walds test) from univariable and multivariable analyses. Only food items to which at least 50% of the cases were exposed are included in the table.

**Exposure**	**Exposed**			**Unexposed**			**Univariable model**			**Multivariable model**		
	Total	Cases	AR %	Total	Cases	AR %	RR	95 % CI	p value	aRR	95 % CI	p value
Kale salad	32	23	72	20	1	5	14,4	2,1-98,3	<0,001	14.9	1.9-117	0.010
Herb sauce	15	10	67	36	14	39	1,7	1,0-3,0	0,070	1.2	0.4-3.5	0.736
Blue cheese	19	11	58	34	13	38	1,5	0,9-2,7	0,168	1.1	0.4-2.7	0,856
Cold smoked salmon	17	10	59	35	14	40	1,5	0,8-2,6	0,202	1,0	0.3-2.8	0.944
Salmon cheesecake	21	11	52	31	12	39	1,4	0,7-2,5	0,330	0.9	0.3-2.6	0.885
Pickles	5	3	60	44	20	45	1,4	0.6-2.9	0.537	0.9	0.2-3.5	0.881
Smoked salmon	41	19	46	14	5	36	1,3	0,6-2,8	0,489	0.8	0.3-2.5	0.718
Rice pudding	14	7	50	39	16	41	1,2	0,6-2,3	0,561			
Fresh smoked salmon	34	15	44	23	9	39	1,1	0,6-2,1	0,708			
Brie cheese	30	14	47	26	11	42	1,1	0,6-2,0	0,743			
Cold poached salmon	20	10	50	33	15	45	1,1	0,6-2,0	0,748			
Morbier cheese	13	6	46	40	18	45	1,0	0,5-2,0	0,942			
Salmon pate	20	9	45	31	14	45	1,1	0,5-1,9	0,991			
Pickled herring, sauce A	16	7	44	36	16	44	1,0	0,5-1,9	0,963			
Pickled herring, sauce B	28	12	43	27	12	44	1,0	0,5-1,8	0,906			
Candy	27	11	41	26	11	42	1,0	0,5-1,8	0,908			
Beetroot salad	31	14	45	23	11	48	1,0	0,5-1,7	0,846			
Pickled herring, sauce C	20	8	40	35	15	43	0,9	0,5-1,8	0,836			
Pickled cucumber	14	6	43	37	17	46	0,9	0,5-1,9	0,843			
Fig marmalade	14	6	43	38	18	47	0,9	0,5-1,8	0,772			
Pickled herring, sauce D	41	16	39	18	8	44	0,9	0,5-1,7	0,696			

Grapes	15	6	40	37	17	46	0,9	0,4-1,8	0,696			
Whipped cream	24	9	38	30	14	47	0,8	0,4-1,5	0,498			
Pickled herring, sauce E	20	7	35	33	16	48	0,7	0,4-1,4	0,337			
Homemade jam	29	10	34	27	13	48	0,7	0,4-1,4	0,299			
Smoked moose sausage	27	10	37	29	15	52	0,7	0,4-1,3	0,269			
Christmas ham	45	16	36	14	7	50	0,7	0,4-1,4	0,333			
Cheese	25	9	36	28	15	54	0,7	0,4-1,2	0,200			
Cheesecake	24	8	33	32	16	50	0,7	0,3-1,3	0,212			
Pickled herring, sauce F	22	7	32	33	16	48	0,7	0,3-1,3	0,220			
Sausage made with lard	19	6	32	33	16	48	0,7	0,3-1,4	0,235			
Sauce	25	8	32	32	16	50	0,6	0,3-1,3	0,172			
Mushroom omelette	27	9	33	28	15	54	0,6	0,3-1,2	0,130			
Meatballs	52	19	37	10	6	60	0,1	0,3-1,1	0,166			
Ribs	21	7	33	31	17	55	0,6	0,3-1,2	0,127			
Red cabbage	21	6	29	35	17	49	0,6	0,3-1,3	0,141			
Ice chocolate	14	4	29	36	18	50	0,6	0,2-1,4	0,171			
Butter	31	10	32	23	13	57	0,6	0,3-1,1	0,075			
Pickled herring, sauce G	38	12	32	20	12	60	0,5	0,3-1,0	0,037			
Roast beef	30	9	30	28	16	57	0,5	0,3-1,0	0,037			
Pickled herring, sauce H	15	4	27	37	19	51	0,5	0,2-1,3	0,104			
Raw spiced salmon	33	9	27	24	14	58	0,5	0,2-0,9	0,018			
Smoked deer meat	30	8	27	26	15	58	0,5	0,2-0,9	0,019			
Mustard	42	13	31	16	11	69	0,5	0,3-0,8	0,009			
Potato and fish gratin	39	11	28	19	12	63	0,5	0,2-0,8	0,011			
Onion sausage	17	4	24	36	20	56	0,4	0,2-1,1	0,029			
Fruit	14	3	21	37	19	51	0,4	0,2-1,2	0,054			
Cheddar cheese	14	3	21	39	21	54	0,4	0,1-1,1	0,037			

Small sausages	40	11	28	18	13	72	0,4	0,2-0,7	0,001			

Supplementary Table 4. Total number, number of cases and attack rate (AR) for individuals exposed and unexposed to different dishes served at the dinner 13th of December in Stockholm County, Sweden 2019, risk ratio (RR) with 95% confidence interval (CI) and p-value (Walds test) from univariable and multivariable analyses. Only food items to which at least 50% of the cases were exposed to, are included in the table.

	**Exposed**			**Unexposed**			**Univariable model**			**Multivariable model**		
**Exposure**	Total	Cases	AR %	Total	Cases	AR %	RR	95 % CI	p value	aRR	95 % CI	p value
Kale salad	27	19	70	5	1	20	3.5	0.6-21.3	0.17	4.0	0.9-18.0	0.067
Cured salmon	20	14	70	15	5	33	2.1	1,0-5,0	0.06	2.6	1.4-4.8	0.002
Hard rye bread	14	11	79	20	8	40	2,0	1.1-3.6	0.03	2.3	1.4-3.8	0.002
Pickled beets	19	13	68	11	4	36	1.9	0.8-4.4	0.15	NA*		
Red cabbage salad	16	11	69	10	4	40	1.7	0.7-4.0	0.21	NA		
Pannacotta	29	18	62	8	3	38	1.7	0.6-4.3	0.30	NA		
Smoked salmon	27	16	59	8	3	38	1.6	0.6-4.1	0.35	NA		
Chocolate mousse	26	16	62	10	4	40	1.5	0.7-3.5	0.31	NA		
Sushi	25	15	60	9	3	33	1.4	0.6-3.0	0.47	NA		
Pickled herring	20	11	55	13	7	54	1.0	0.5-2,0	0.95	NA		
Sour dough	18	10	56	14	8	57	1,0	0.5-1.8	0.93	NA		
Venison fillet	26	14	54	7	4	57	0.9	0.5-2,0	0.88	NA		
Butter	30	17	57	6	4	67	0.9	0.4-1.6	0.63	NA		
Eggs and prawns in mayonnaise	24	11	46	7	4	57	0.8	0.4-1.8	0.58	NA		
Meatballs	25	13	52	10	7	70	0.7	0.4-1.3	0.30	NA		

*Effect modification was identified in the stratified analysis of pickled beets (strata by exposure of kale salad) and pickled beets was therefore excluded from the multivariable analysis.
